# Complex systems analysis of bladder cancer susceptibility reveals a role for decarboxylase activity in two genome-wide association studies

**DOI:** 10.1186/s13040-016-0119-z

**Published:** 2016-12-12

**Authors:** Samantha Cheng, Angeline S. Andrew, Peter C. Andrews, Jason H. Moore

**Affiliations:** 1 Department of Biostatistics and Epidemiology, Institute for Biomedical Informatics, Perelman School of Medicine, University of Pennsylvania, Philadelphia, PA 19104-6116 USA; 2 Department of Epidemiology, Geisel School of Medicine, Dartmouth College, Hanover, NH 03755 USA

## Abstract

**Background:**

Bladder cancer is common disease with a complex etiology that is likely due to many different genetic and environmental factors. The goal of this study was to embrace this complexity using a bioinformatics analysis pipeline designed to use machine learning to measure synergistic interactions between single nucleotide polymorphisms (SNPs) in two genome-wide association studies (GWAS) and then to assess their enrichment within functional groups defined by Gene Ontology. The significance of the results was evaluated using permutation testing and those results that replicated between the two GWAS data sets were reported.

**Results:**

In the first step of our bioinformatics pipeline, we estimated the pairwise synergistic effects of SNPs on bladder cancer risk in both GWAS data sets using Multifactor Dimensionality Reduction (MDR) machine learning method that is designed specifically for this purpose. Statistical significance was assessed using a 1000-fold permutation test. Each single SNP was assigned a *p*-value based on its strongest pairwise association. Each SNP was then mapped to one or more genes using a window of 500 kb upstream and downstream from each gene boundary. This window was chosen to capture as many regulatory variants as possible. Using Exploratory Visual Analysis (EVA), we then carried out a gene set enrichment analysis at the gene level to identify those genes with an overabundance of significant SNPs relative to the size of their mapped regions. Each gene was assigned to a biological functional group defined by Gene Ontology (GO). We next used EVA to evaluate the overabundance of significant genes in biological functional groups. Our study yielded one GO category, carboxy-lysase activity (GO:0016831), that was significant in analyses from both GWAS data sets. Interestingly, only the gamma-glutamyl carboxylase (GGCX) gene from this GO group was significant in both the detection and replication data, highlighting the complexity of the pathway-level effects on risk. The GGCX gene is expressed in the bladder, but has not been previously associated with bladder cancer in univariate GWAS. However, there is some experimental evidence that carboxy-lysase activity might play a role in cancer and that genes in this pathway should be explored as drug targets. This study provides a genetic basis for that observation.

**Conclusions:**

Our machine learning analysis of genetic associations in two GWAS for bladder cancer identified numerous associations with pairs of SNPs. Gene set enrichment analysis found aggregation of risk-associated SNPs in genes and significant genes in GO functional groups. This study supports a role for decarboxylase protein complexes in bladder cancer susceptibility. Previous research has implicated decarboxylases in bladder cancer etiology; however, the genes that we found to be significant in the detection and replication data are not known to have direct influence on bladder cancer, suggesting some novel hypotheses. This study highlights the need for a complex systems approach to the genetic and genomic analysis of common diseases such as cancer.

## Findings

Bladder cancer is a form of cancer that typically starts in the inner lining of the bladder, called the urothelium, and can grow into or through other layers of tissue. It is a disease responsible for approximately 16,000 deaths per year, with particular impact in the American male population. Bladder cancer is the fourth most common cancer in men, and affects 1 in 26 men and 1 in 90 women [1]. According to the NHGRI-EBI catalog of published genome-wide association studies (GWAS) [2], there are five single locus genetic variants associated with bladder cancer at a genome-wide significance level. Collectively, these genetic factors explain a very small proportion of the overall risk. The goal of this study was to employ a bioinformatics approach to GWAS analysis that considers pairwise genetic interactions among SNPs followed by gene-level and pathway-level gene set enrichment analyses. This approach is based on the hypothesis that evolution works to stabilize health by building highly redundant gene interaction networks within and between pathways [3]. The result of this complex biology is that the healthy state is resilient to the effects of single mutations. What we observe in common diseases is the accumulation of multiple mutations within these pathways that disrupts their stability and impairs their normal function. This dependence on multiple mutations can be observed as epistasis or non-additive gene-gene interaction [4]. The results of univariate GWAS are consistent with this hypothesis, as few univariate genetic effects have been found that replicate consistently across studies. The goal of our study is to employ a bioinformatics approach to GWAS analysis that is consistent with the idea that some genetic effects will present themselves as genetic interactions that aggregate in genes and pathways [5, 6].

**Fig. 1:**
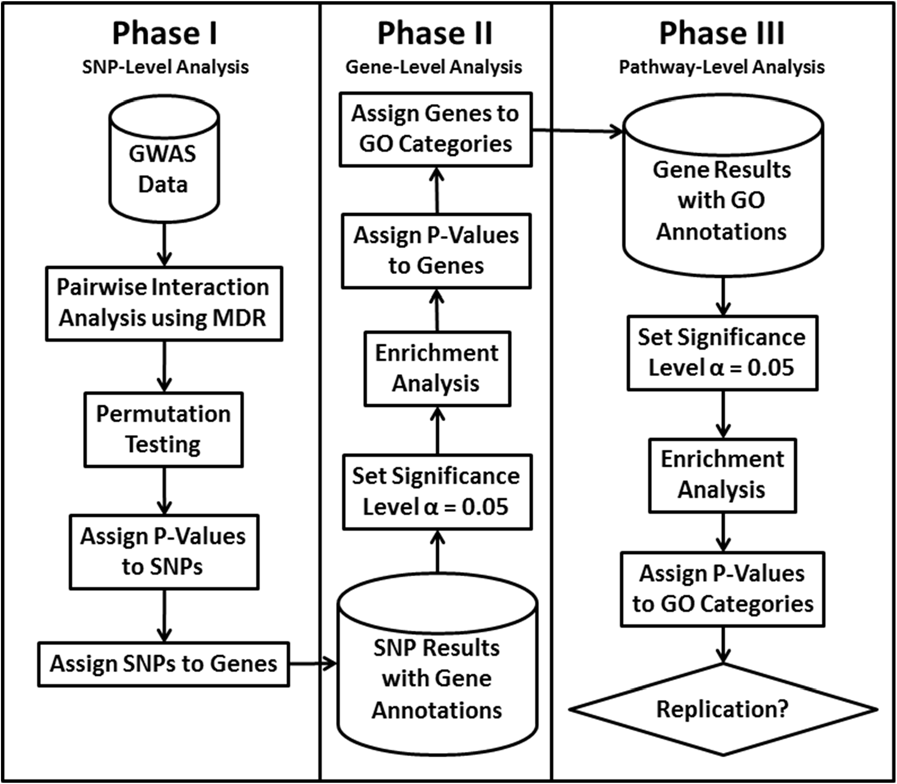
Flowchart summarizing the three phases of our machine learning and gene set enrichment analysis strategy

We briefly present our three-phase analysis approach below and provide a flowchart in Figure 1 as previously presented and applied to GWAS analysis [7, 8]. The GWAS data used is available from the NCBI dbGaP database under accession number phs000346.v2.p2. We used here the two largest GWAS data sets including one with subjects from the U.S.A. and Finland (*n* = 4759; 620901 SNPs) and one from Spain (*n* = 2228, 1072820 SNPs). These were used as detection and replication data sets, respectively. We analyzed only SNPs common to both data sets.

### Phase I: SNP-level analysis

The goal of the first phase is to carry out a pairwise analysis of all SNPs using the Multifactor Dimensionality Reduction (MDR) method to search for additive and non-additive genetic associations [9, 10]. MDR is a nonparametric, genetic model-free machine learning method that collapses high-dimensional genetic data into a single dimension through a process called constructive induction [10]. More information about MDR and its implementation can be found here [11]. We exhaustively evaluated all pairs of SNPs and then assessed statistical significance using a 1000-fold permutation test as described previously [11]. Each SNP was assigned the *p*-value of its strongest pairwise association.

### Phase II: gene-level analysis

At the gene level, we first mapped all SNPs to genes using a window of 500 kb upstream and downstream from the gene start and stop sites in order to capture regulatory variants. We then performed a gene set enrichment analysis to determine if there were more SNPs with p-values at or below the 0.05 significance level in gene regions than would be expected given their size. This was accomplished using a right-tailed Fisher’s exact test implemented in Exploratory Visual Analysis (EVA) [12, 13]. These *P*-values for SNP overabundance were then assigned to each gene.

### Phase III: pathway-level analysis

The final step was to determine whether the genes with a statistically significant overabundance of SNPs aggregate in functional groups defined by Gene Ontology (GO) [14] provided by the Molecular Signatures Database (MSigDB) [15]. This analysis was performed using EVA as described above. *P*-values from the Fisher’s exact test were assigned to each GO category. Replication at the 0.05 significance level was assessed across the two GWAS data sets. As discussed by Kim et al. [7], permutation testing combined with this multistep process with replication helps address false-positives due to multiple testing.

## Results and discussion

Our pathway-level gene set enrichment analysis yielded one GO category, carboxy-lysase activity (GO:0016831), that was significant in both GWAS data sets with *p*-values of 0.023 and 0.043. This pathway is sometimes referred to as decarboxylase activity and has previously been explored as a target for chemotherapy because of its role in polyamine metabolism that is required for tumor growth [16]. Little is known about the direct role of this pathway in bladder cancer and none of the genes from this pathway have been implicated from the results of univariate GWAS analyses. As such, our results are novel and suggest this pathway and its genes as a new biological hypothesis for bladder cancer genetic susceptibility. If validated, genes in this pathway could be targets for therapy given the current focus on polyamines for chemotherapy.

There were five significant genes in the detection data set and three genes in the replication data set that are members of the carboxy-lysase GO pathway. One of these, gamma-glutamyl carboxylase (GGCX), was significant with p-values of 0.03 and 0.04 in the detection and replication data sets, respectively. The GGCX protein is an enzyme that catalyzes post-translational modifications to a vitamin K-dependent protein that functions in coagulation. Mutations in GGCX are typically associated with combined deficiency of vitamin K-dependent clotting factors 1 and hemorrhagic disease [17]. Although there is no evidence this gene plays a role in bladder cancer, it has been associated with prostate cancer in several GWAS [18, 19]. It is possible that genetic variation in this gene is also a risk factor for bladder cancer, but that synergistic interactions between multiple SNPs are necessary to observe a phenotype. Importantly, there are several drug-GGCX interactions according to The Drug Gene Interaction Database (DGIdb) [20]. Anisindione is one such drug and, according to the NCBI PubChem database [21], is a synthetic anticoagulant that disrupts the synthesis of clotting factors leading to the inhibition of gamma-carboxylation of glutamic acid. The repositioning of GGCX-related drugs for the treatment of bladder cancer is an open question. Indeed, these drugs are often considered for anticoagulation treatment in cancer patients because of their beneficial effect on the tumor microenvironment [22].

It is worth noting that the branched chain keto acid dehydrogenase E1 alpha (BCKDHA) and beta (BCKDHB) gene forms were significant in the detection and replication data, respectively. Although these two genes are on different chromosomes, their protein products are part of the same complex that is involved in the catabolism of several amino acids. The BCKD complex is comprised of three catalytic components, one of them being E1, a heterotetramic branched-chain alpha-keto acid decarboxylase. BCKDHA codes for the alpha subunit of E1, while BCKDHB codes for the beta subunit of E1. Without this decarboxylase component, 2-oxycarboxylic acid accumulates in blood and tissues, and causes maple syrup urine disease [23–25]. There is no known link between the effects of the BCKD complex and bladder cancer, but univariate GWAS have implicated BCKDHA in prostate [26] and colorectal [27] cancer.

There were several limitations of the present study that should be kept in mind when interpreting the results. First, power to detect gene-gene interactions is always a concern given the curse of dimensionality that comes with considering multilocus genotype combinations. We addressed the power concerns by using the MDR machine learning method and by using a liberal significance level of 0.05. We also used the two largest GWAS data sets available through dbGaP. Despite these measures, power may still be a limiting issue. Second, we did not correct for the correlation structure of the SNPs in the gene regions. This was primarily due to the potential co-occurrence of interactions and linkage disequilibrium due to selection. Consideration of correlation is more complicated for gene-gene interactions studies than it is for significance testing of single SNPs using univariate methods. Third, the detection and replication GWAS data were primarily Caucasian subjects from the U.S., Finland, and Spain. These results may not generalize to other populations of different ethnic background. In fact, we fully expect the spectrum of gene-gene interactions to shift from population to population as genetic architecture changes due to different genetic backgrounds and local ecologies. We also expect statistical measures of interaction to change as allele frequencies change. These factors represent significant challenges to detecting and characterizing gene-gene interactions. This study partly addresses some of these concerns by focusing on the aggregation of statistical results at the pathway level thus taking the focus off inferential statistical analysis at the SNP level.

It is also important to note that the use of a more liberal significance threshold to improve power means that there is a higher type I error rate that could lead to more false-positives. This should also be taken into consideration before deciding to carry out a confirmatory study.

The results of this study support the idea that a bioinformatics approach to GWAS analysis of bladder cancer yields novel, replicable results not discovered using univariate statistical methods. As we exhaust efforts to identify and list SNPs that have universal main effects on disease risk across genetic backgrounds and local ecologies, it will be important explore alternative bioinformatics methods that are designed to embrace a complex genetic architecture underlying most common diseases such as cancer. This means we need methods that are able to capture genetic effects that are dependent on genetic background, environmental context, or that might be based on genetic variants that segregate only in a small number of families giving rise to locus heterogeneity. This study is one of the first to measure gene-gene interactions on a genome-wide scale and to measure their aggregation across biochemical pathways and functional groupings of genes as detailed in GO. The carboxy-lysase activity pathway revealed by this approach was identified across two different GWAS data sets and raises the question as to whether decarboxylase genes should be investigated as drug targets. These results are preliminary and warrant confirmatory studies.
